# Exposure to Ultraviolet (UV-C) Radiation Increases Germination Rate of Maize (*Zea maize* L.) and Sugar Beet (*Beta vulgaris*) Seeds

**DOI:** 10.3390/plants8020049

**Published:** 2019-02-24

**Authors:** Pouria Sadeghianfar, Meisam Nazari, Gunter Backes

**Affiliations:** 1 Department of Crop Sciences, Faculty of Agricultural Sciences, Georg-August University of Göttingen, Büsgenweg 5, 37077 Göttingen, Germany; meisam.nazari@stud.uni-goettingen.de; 2 Department of Organic Plant Breeding and Agrobiodiversity, Faculty of Organic Agricultural Sciences, University of Kassel, Nordbahnhofstr. 1a, 37213 Witzenhausen, Germany; gbackes@uni-kassel.de

**Keywords:** germination rate, maize, sugar beet, UV-C

## Abstract

This study investigated the effect of ultraviolet (UV-C) radiation on the germination percentage, germination rate, radicle length, and plumule length of maize and sugar beet seeds. The experiment was implemented in six replicates of 30 seeds per replicate and in sterilized petri dishes under laboratory conditions. Treatments included UV-C (254 nm) radiation exposure durations of 0 min (control), 30 min, 2 h, 4 h, 8 h, and 12 h. The UV-C radiation treatments did not significantly affect the germination percentage of the seeds (*p* < 0.05). However, the seeds germination rate was significantly affected by the UV-C radiation treatments. The treatments of 8 h and 12 h exposure duration led to the highest seed germination rates in maize and sugar beet, respectively. Lowest seed germination rates belonged to the controls. The radicle length of maize seeds was significantly affected by the UV-C radiation treatments, but the treatments did not significantly affect the radicle length of sugar beet seeds. The 12 h exposure to UV-C radiation treatment resulted in the largest radicle in maize, which was 2.08 cm larger than the radicle of the control seeds. The UV-C radiation treatments had a statistically significant effect on the plumule length of maize and sugar beet seeds. The treatment 8 h UV-C exposure duration led to the largest plumule in maize and sugar beet, which were 0.32 cm and 0.83 cm larger than the plumule of the control seeds, respectively. Breaking down the seed coat and increasing the temperature by UV-C radiation are potential reasons for the observed positive effects.

## 1. Introduction

Plants use dormancy mechanisms to postpone seed germination until favorable conditions are provided. Breaking seed dormancy is necessary for the production of important crops. Various approaches have been used to overcome seed dormancy, such as salinity and temperature [1], scarification [2], regulatory hormones [3], fungal inoculation [4], chemical [5], and ultrasound [6]. 

Increased global ultraviolet (UV) radiation due to the depletion of the stratospheric ozone is an important concern [7]. There are three types of UV radiation: UV-A (315–390 nm), UV-B (280–315 nm), and UV-C (100–280 nm). UV-C radiation can affect microorganisms and plants. UV-C radiation damages the DNA of bacteria, viruses, and other pathogens and destroys their ability to multiply and cause disease [8]. The physiological and biochemical processes of plants can be affected by UV-C radiation [9]. Leaf chlorophyll, protein content, and peroxidase enzyme activity in plants can also be affected by UV-C radiation [10]. Siddiqui et al. [11] demonstrated that the pretreatment of groundnut (*Arachis hypogaea* L.) and mung bean (*Vigna radiata* L.) seeds by UV-C enhanced the weight of shoot and root. UV-C radiation was also shown to increase the germination percentage of the groundnut [12]. In contrast, the exposure of Andean lupin (*Lupinus mutabilis*) to UV-C radiation reduced its germination percentage [10]. Nonetheless, Shetta et al. reported that UV-C radiation did not significantly affect the germination percentage of *Acacia ampliceps* seeds [13]. These contradictory results might be due to the use of different plant species and UV-C exposure durations. More research is necessary to investigate the effect of UV-C radiation on seed germination, as only a few studies have been implemented in this area.

Sugar beet (*Beta vulgaris*) and maize (*Zea maize* L.) are two economically important plants. Rapid and uniform seedling emergence in sugar beet increases the probability of high yields. However, low soil temperature can delay the emergence of sugar beet seedlings and expose them to soil crusting and seedling diseases [14]. For both the sugar beet and maize, rapid and uniform seedling establishment are important for achieving high yields [15]. This study investigated the effect of UV-C radiation on the germination percentage, germination rate, radicle length, and plumule length of sugar beet and maize seeds.

## 2. Materials and Methods

Seeds were provided by KWS SAAT SE (Einbeck, Germany). The varieties were Figaro for maize and Evamaria for sugar beet. Mature and undamaged seeds of the same size were selected and disinfected by soaking in NaOH 1% solution for 10 min. Seeds were then rinsed with distilled water three times.

The experiment was implemented in six replicates of 30 seeds per replicate in sterilized petri dishes under laboratory conditions. The seeds of each replicate were independently exposed to UV-C radiation for different durations. Treatments included UV-C radiation exposure durations of 0 min (control), 30 min, 2 h, 4 h, 8 h, and 12 h. The UV-C wavelength was 254 nm and the UV-C intensity was 54 mJ cm^−2^. For the UV-C radiation treatments, seeds in petri dishes filled with 5 mL distilled water were placed in the UV-C apparatus (PCR UV^3^ HEPA Workstation, Analytik Jena AG). The distance of seeds from the UV-C radiation lamps was approximately 50 cm. After exposure to UV-C radiation, the seeds were placed on Whatman No. 1 filter paper moistened with tap water in sterilized petri dishes. Germination tests were carried out as a completely randomized design (CRD) at room temperature (24 °C) in darkness. The evaluated variables for assessing the effect of UV-C radiation included germination percentage, germination rate, radicle length, and plumule length. A seed was considered germinated when the tip of the radicle had grown 1 mm out of the seed coat [16]. The germination percentage was calculated by dividing the number of germinated seeds by the total number of seeds multiplied by 100. The number of germinated seeds was recorded at 10-hour intervals for 80 and 140 hours for maize and sugar beet, respectively. The germination rate was calculated according to Maguire [17] as:

(1) $$GR=\;\frac{X_{1}}{Y_{1}}+\frac{X_{2}}{Y_{2}}+\ldots +\frac{X_{n}}{Y_{n}},$$

where *X*_1_, *X*_2_, and *X_n_* are the number of seeds germinated on the first, second, and nth hour, respectively, and *Y*_1_, *Y*_2_, and *Y_n_* are the number of hours from sowing to first, second, and nth count, respectively.

A precise ruler was used to measure the radicle length and plumule length of the seeds. The length of seed radicle and plumule for each species and treatment was measured after 80 hours for maize and after 140 hours for sugar beet.

The data were analyzed by SPSS version 23.0 (Statistical Package for Social Sciences, IBM Inc., Chicago, USA). Normality and variances equality were checked before data analysis by the Shapiro-Wilk test and Leven’s test, respectively. The data not fulfilling these two prerequisites were ln-transformed. A one-way ANOVA was used to check the significance of the treatments (*p* < 0.05). Tukey’s HSD (honestly significant difference) was used to compare the arithmetic means of the variables (*p* < 0.05).

## 3. Results

**Figure 1:**
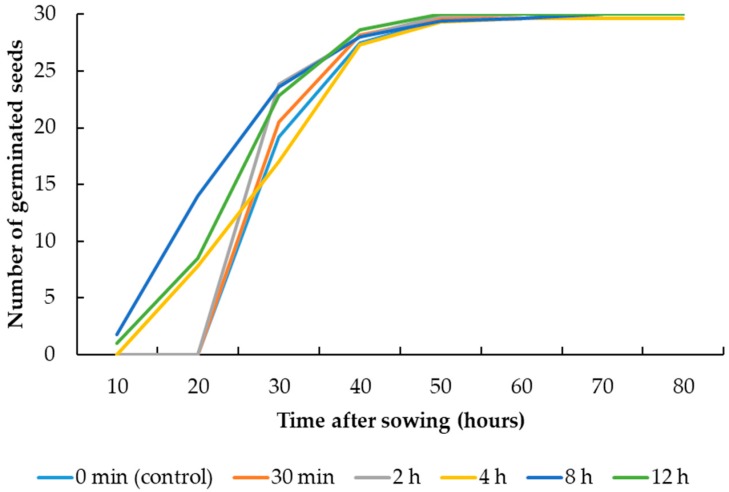
Cumulative seed germination of maize affected by the UV-C radiation treatments.

**Figure 2:**
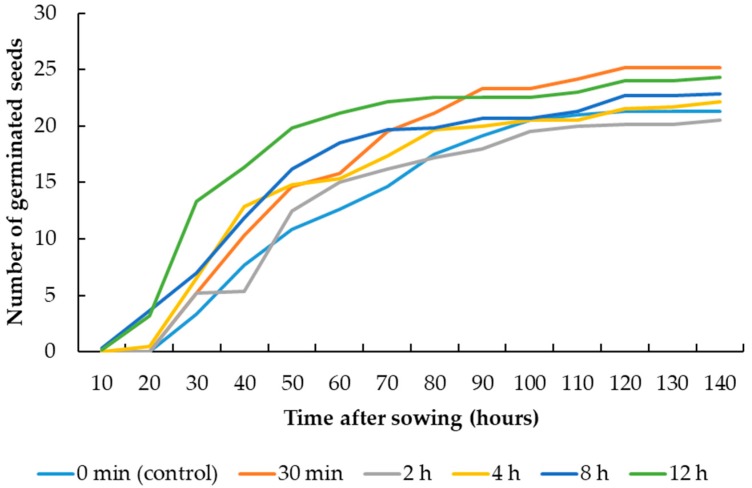
Cumulative seed germination of sugar beet affected by the UV-C radiation treatments.

The UV-C radiation treatments did not significantly affect the germination percentage of maize and sugar beet seeds (Table 1). However, the germination rate of maize and sugar beet seeds was significantly affected by the UV-C radiation treatments. The treatments 8 h and 12 h exposure duration led to highest seed germination rates in maize (4.16) and sugar beet (3.61), respectively (Table 2). Lowest seed germination rates belonged to the controls. Figure 1 and Figure 2 show the cumulative seed germination of maize and sugar beet, which also graphically indicate the higher germination rates of maize and sugar beet seeds affected by the 8 h and 12 h UV-C radiation treatments, respectively, compared with the controls. The radicle length of maize seeds was significantly affected by the UV-C radiation treatments, but the treatments did not significantly affect the radicle length of sugar beet seeds. The 12 h exposure to UV-C radiation treatment resulted in the largest radicle (5.88 cm) in maize, which was 2.08 cm larger than the radicle of the control. The UV-C radiation treatments had a statistically significant effect on the plumule length of maize and sugar beet seeds (Table 1). The treatment consisting of an 8 h UV-C exposure duration led to the largest plumule length in maize (1.01 cm) and sugar beet (4.18 cm), which were 0.32 cm and 0.83 cm larger than the plumule of the controls, respectively.

## 4. Discussion

The germination percentage of the investigated seeds was not significantly changed by the treatments. However, the seeds germination rate, radicle length (except that of sugar beet), and plumule length were significantly increased by the UV-C exposure treatments. [11] showed that the exposure of groundnut (*Arachis hypogaea* L.) and mung bean (*Vigna radiata* L.) seeds to UV-C enhanced the shoot and root weights. UV-C radiation also increased the germination percentage of groundnut [12]. UV-C (100–280 nm) photons have higher energy than visible light (> 400 nm) photons, and therefore can affect plant cells more strongly [18]. In this study, the positive effect of UV-C radiation on the seeds might have been due to three major processes: (1) UV-C radiation broke down the seed coat, resulting in higher and faster oxygen and water imbibition by the seeds and alleviated dormancy; (2) UV-C radiation increased temperature and accelerated the provision of the optimum temperatures required for germination; (3) Increased temperature by UV-C radiation increased the seed respiration and mitochondrial activities. All assumptions could contribute an unknown share to the observed results.

## 5. Conclusions

This study investigated the effect of UV-C radiation on the germination percentage, germination rate, radicle length, and plumule length of sugar beet and maize seeds. UV-C radiation was indicated to positively affect the germination rate, radicle length (except that of sugar beet), and plumule length of the seeds. A breakdown in seed coat and an increase in temperature by UV-C radiation are potential reasons for the observed positive effects.

## Figures and Tables

**Table 1:** Analysis of variance results of the effect of the UV-C radiation treatments on the seeds germination percentage, germination rate, radicle length, and plumule length (one-way ANOVA, *p* < 0.05); ns and * indicate statistically non-significant and significant differences between the treatments, respectively.

Species/Variables	df	Sum of Squares	Mean Square	F-Value	p-Value
*Maize*					
Germination percentage	5	6.16	1.23	2.5	0.052 ns
Germination rate	5	4.21	0.84	15.83	0.000 *
Radicle length	5	20.09	4.01	12.21	0.000 *
Plumule length	5	1.32	0.26	4.19	0.005 *
*Sugar beet*					
Germination percentage	5	1050.68	210.13	1.56	0.200 ns
Germination rate	5	7.69	1.53	5.46	0.001 *
Radicle length	5	5.14	1.03	6.33	0.082 ns
Plumule length	5	15.23	3.04	3.63	0.011 *

**Table 2:** Comparison of the arithmetic means of the seeds germination percentage, germination rate, radicle length, and plumule length affected by the UV-C radiation treatments (Tukey’s HSD, *p* < 0.05). Different letters within a column show a statistically significant difference.

Species/Treatment	Germination Percentage (%)	Germination Rate	Radicle Length (cm)	Plumule Length (cm)
*Maize*				
0 min (control)	100 a	3.21 b	3.80 c	0.69 ab
30 min	100 a	3.28 b	3.70 c	0.50 b
2 h	100 a	3.39 b	4.54 bc	0.59 ab
4 h	98.8 a	3.51 bc	5.11 ab	0.94 ab
8 h	100 a	4.16 a	4.67 bc	1.01 a
12 h	100 a	3.90 ac	5.88 a	0.93 ab
CV (± %)	1	11	20	38
*Sugar beet*				
0 min (control)	71.1 a	2.26 b	3.17 a	3.35 b
30 min	83.8 a	2.82 ab	3.13 a	3.06 b
2 h	68.3 a	2.30 b	3.37 a	3.18 b
4 h	73.8 a	2.72 ab	3.81 a	3.65 ab
8 h	76.1 a	3.06 ab	4.60 a	4.18 a
12 h	81.1 a	3.61 a	4.73 a	3.73 ab
CV (± %)	15	24	28	15
